# Isothiocyanate From *Moringa oleifera* Seeds Inhibits the Growth and Migration of Renal Cancer Cells by Regulating the PTP1B-dependent Src/Ras/Raf/ERK Signaling Pathway

**DOI:** 10.3389/fcell.2021.790618

**Published:** 2022-01-04

**Authors:** Jing Xie, Ying-Yan Qian, Yang Yang, Lin-Jie Peng, Jia-Ying Mao, Ming-Rong Yang, Yang Tian, Jun Sheng

**Affiliations:** ^1^ College of Food Science and Technology, Yunnan Agricultural University, Kunming, China; ^2^ Engineering Research Center of Development and Utilization of Food and Drug Homologous Resources, Ministry of Education, Yunnan Agricultural University, Kunming, China; ^3^ National R&D Center for Moringa Processing Technology, Yunnan Agricultural University, Kunming, China; ^4^ College of Science, Yunnan Agricultural University, Kunming, China; ^5^ Yunnan Provincial Engineering Research Center for Edible and Medicinal Homologous Functional Food, Yunnan Agricultural University, Kunming, China; ^6^ Key Laboratory of Pu-er Tea Science, Ministry of Education, Yunnan Agricultural University, Kunming, China

**Keywords:** *Moringa oleifera*, isothiocyanate, renal cancer cells, cell growth and migration, PTP1B, Src/Ras/Raf/ERK signaling pathway

## Abstract

*Moringa oleifera* Lam. is a tropical and subtropical plant that has been used for centuries as both food and traditional medicine. 4-[(α-L-Rhamnosyloxy) benzyl] isothiocyanate (MIC-1) is an active substance in *M. oleifera*, with anti-cancer activity. However, whether MIC-1 exerts anti-renal cancer effects is unknown. Therefore, the aim of the present study was to evaluate the effects of MIC-1 on the growth and migration of renal cell carcinoma (RCC) cells and to identify the putative underlying mechanism. We found that, among 30 types of cancer cells, MIC-1 exerted the strongest growth inhibitory effects against 786-O RCC cells. In addition, MIC-1 (10 μM) significantly inhibited the growth of five RCC cell lines, including 786-O, OSRC-2, 769-P, SK-NEP-1, and ACHN cells, but was not toxic to normal renal (HK2) cells. Also, MIC-1 suppressed 786-O and 769-P cell migration and invasion abilities, and reduced the expression of matrix metalloproteinase (MMP)-2 and MMP-9. Furthermore, MIC-1 induced apoptosis and cell cycle arrest, increased Bax/Bcl-2 ratio, and decreased cell cycle-related protein expression in 786-O cells and 769-P cells. Molecular docking and small-molecule interaction analyses with PTP1B both showed that MIC-1 inhibited PTP1B activity by binding to its active site through hydrogen bonding and hydrophobic interactions. Additionally, MIC-1 could suppress the growth and migration of 786-O cells by inhibiting PTP1B-mediated activation of the Src/Ras/Raf/ERK signaling pathway. *In vivo* experiments further showed that MIC-1 markedly inhibited the growth of xenograft tumors in mice, and greatly increased Bax/Bcl-2 ratio in tumor tissues. In addition, MIC-1 had no effect on the PTP1B-dependent Src/Ras/Raf/ERK signaling pathway in HCT-116 cells, Hep-G2 cells, and A431 cells. Overall, our data showed that MIC-1 could be a promising, non-toxic, natural dietary supplement for the prevention and treatment of renal cancer.

## Introduction

Renal cell carcinoma (RCC) is a common and deadly disease with a worldwide estimate for 2020 of ∼431,000 new cases and ∼179,000 deaths (Global Cancer Observatory). RCC originates from the epithelial cells of the renal convoluted tubule and is one of the most commonly diagnosed malignant tumors of the urinary system. RCC accounts for 80–90% of all renal malignancies, and has an incidence rate of approximately 2.1% among systemic tumors (Siegel et al., 2018). Additionally, mostly due to smoking and dietary changes, the incidence of RCC has gradually increased in recent years. Although many targeted drugs are available for the treatment of RCC, a high recurrence rate and drug resistance remain the biggest challenges facing RCC patients (Ebos et al., 2009).

Protein-tyrosine phosphatase 1B (PTP1B, also known as PTPN1), a nonreceptor type phosphatase with oncogenic properties, is involved in growth factor signaling pathways (Bollu et al., 2017). Numerous studies have shown that PTP1B is an important target for antitumor therapy and is closely related to the occurrence and development of breast cancer, liver cancer, colon cancer, non-small cell lung cancer, prostate cancer, and pancreatic cancer (Kostrzewa et al., 2019). PTP1B has been reported to exert its oncogenic activities through the activation of the nonreceptor tyrosine kinase Src (Zhang and Yu, 2012), which can enhance Ras/Raf/ERK/PI3K/mTOR pathway signaling and thereby promote tumor cell proliferation and metastasis (Penuel and Martin, 1999; Bollu et al., 2017). A previous study reported that the mRNA expression of Src was high in RCC samples compared with that in normal kidney samples (Majid et al., 2011). In addition, there is some evidence to suggest that Src may be associated with the malignancy of RCC cells and the poor prognosis of RCC patients, and the inhibition of Src may represent a promising option for the treatment of RCC (Yonezawa et al., 2005; Roseweir et al., 2016).

*Moringa oleifera* Lam. is a tree of the Moringaceae family found abundantly in many tropical and subtropical countries. Its leaves, seeds, bark, roots, sap, and flowers are widely used in traditional medicine (Stohs and Hartman, 2015). *M. oleifera* seeds are rich in nutrients such as proteins, lipids, and vitamins, and also contain flavonoids, phenolic acids, alkaloids, glucosinolates, isothiocyanates, and thiocarbamates, as well as other biologically active substances (Leone et al., 2016). Additionally, *M. oleifera* seeds are reported to possess anti-inflammatory (Mahajan et al., 2007), antitumor (Asaduzzaman et al., 2018), hypoglycemic (Al-Malki and El Rabey, 2015), hypolipidemic (Mapfumo et al., 2019), hypotensive (Randriamboavonjy et al., 2016), and liver-protective (Hamza, 2010) properties.

**FIGURE 1:**
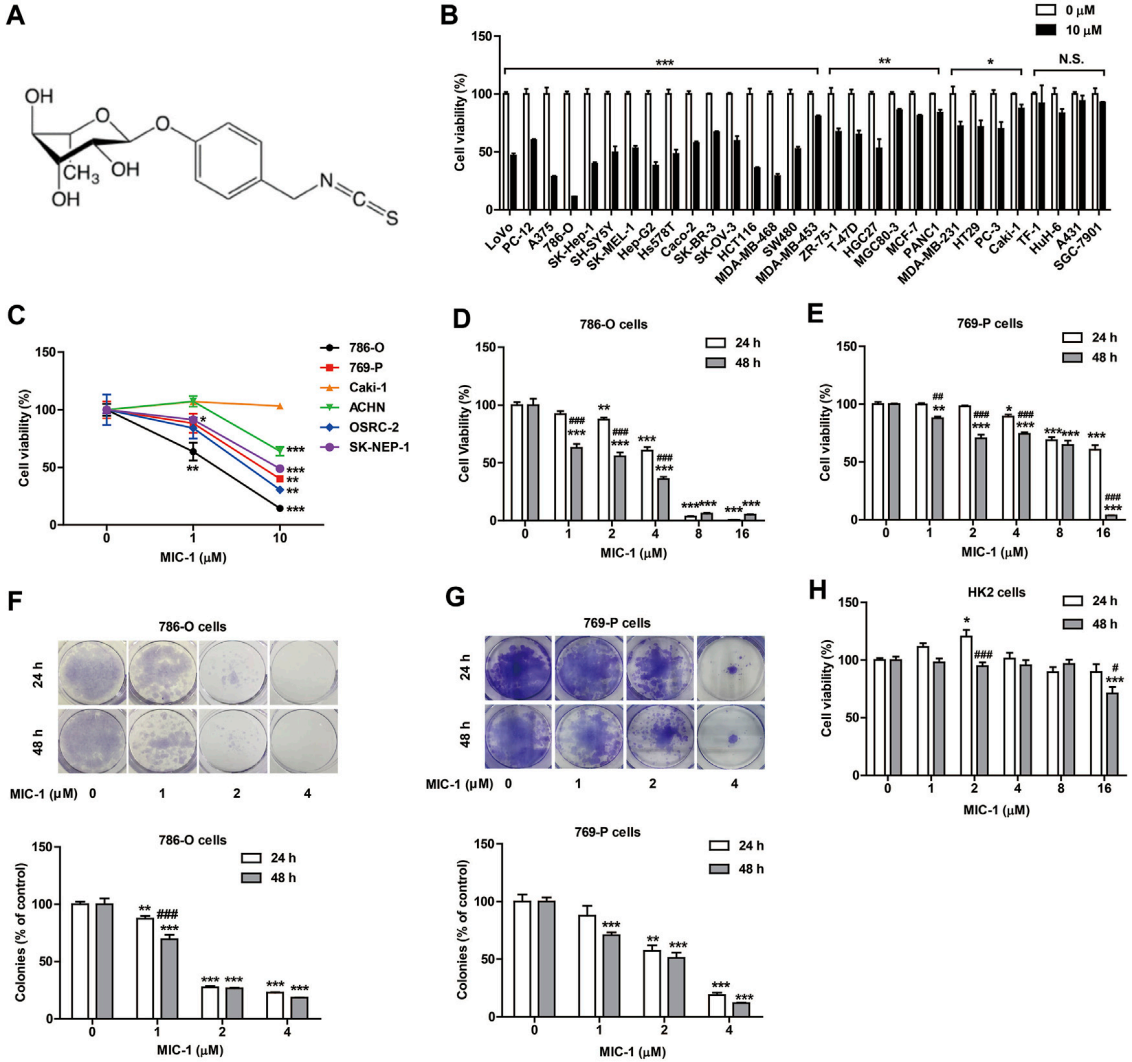
MIC-1 inhibits the growth of RCC cells. **(A)** The molecular structure of MIC-1. **(B)** The viability of 30 types of cancer cells following MIC-1 (0 or 10 μM) treatment for 48 h. **(C)** The viability of six types of renal cancer cells following MIC-1 (0, 1, or 10 μM) treatment for 48 h. The viability of 786-O cells **(D)** and 769-P cells **(E)** treated with different concentrations of MIC-1 (0, 1, 2, 4, 8, or 16 μM) for 24 or 48 h. Clonogenic ability of 786-O cells **(F)** and 769-P **(G)** was analyzed by a colony formation assay. Cells were incubated with MIC-1 (0, 1, 2, or 4 μM) for 24 or 48 h. **(H)** The viability of HK2 cells treated with different concentrations of MIC-1 (0, 1, 2, 4, 8, or 16 μM) for 24 or 48 h. Values represent the means ± SEM from three independent experiments and statistical analysis was performed by unpaired two-tailed Student’s t tests, one-way ANOVA, or two-way ANOVA; *p < 0.05, **p < 0.01, ***p < 0.001: MIC-1 compared with the control (0 μM); ^#^ *p <* 0.05, ^##^ *p <* 0.01, ^###^ *p <* 0.001: 48 h compared with 24 h at the same MIC-1 concentration.

Isothiocyanates (ITCs) are sulfur-containing plant secondary metabolites widely present in *Brassica* plants (McNaughton and Marks, 2003). Emerging evidence has shown that the leaves and seeds of *M. oleifera* are rich in ITCs, which are among its main active ingredients (Dhakad et al., 2019). *M. oleifera* isothiocyanates (MITCs) contain the same pharmacophore (R–N=C=S) as ITCs from other *Brassica* plants, but differ due to the presence of an aromatic ring and rhamnose moiety (Waterman et al., 2015). There are four unique ITCs in *M. oleifera*, all with strong biological activity, with 4-[(α-L-rhamnosyloxy) benzyl] isothiocyanate (MIC-1; Figure 1A) being the most abundant (Jaja-Chimedza et al., 2018). Numerous studies have shown that MIC-1 exerts hypoglycemic (Jaja-Chimedza et al., 2018), hypolipidemic (Waterman et al., 2015), anti-inflammatory (Waterman et al., 2014; Cheng et al., 2019), antioxidant (Tumer et al., 2015), and anticancer (Brunelli et al., 2010; Rajan et al., 2016; Fahey et al., 2018) effects. However, whether MIC-1 possesses anti-RCC activity remains unknown.

The aim of the present study was to evaluate the inhibitory effects of MIC-1 on the growth and migration of RCC cells and to elucidate the possible mechanisms of action involved.

## Materials and Methods

### Preparation of MIC-1

*M. oleifera* seeds (Yunnan Tianyou Technology Development Co., Ltd; Dehong, Yunnan, China) were hulled, crushed, and passed through a 30-mesh sieve. The resulting *M. oleifera* seed powder was processed using a Soxhlet extractor. The powder was sealed, petroleum ether was added for deoiling, and the product was heated to reflux for more than 12 h until deoiling was complete. Ten grams of dry deoiled *M. oleifera* seed powder was added to a glass beaker, purified water was added at a solvent ratio of 1:60 (g/ml), the pH was adjusted to 5 with acetic acid, and then the powder was placed in a water bath at 30°C with mechanical stirring for 9 h, and finally centrifuged (4,000 rpm, 5 min) to collect the supernatant. The supernatant was extracted three times with 1:3 volume of dichloromethane, all fractions were combined, and then concentrated under reduced pressure with a rotary evaporator at 30°C, and protected from light until a white solid was produced. The white solid was subjected to recrystallization with dichloromethane. After five recrystallizations, MIC-1 with a purity of 98% was obtained, and the total yield was 4.70%. The resulting MIC-1 was analyzed by ^1^H-^13^C nuclear magnetic resonance spectroscopy and high-resolution mass spectrometry. The data obtained by the analysis were consistent with those previously reported (Gueyrard et al., 2010) and the structure of MIC-1 was confirmed to be correct (Figure 1A).

### Cell Culture

Thirty-four cancer cell lines (LoVo, PC-12, A375, 786-O, SK-Hep-1, SH-SY5Y, SK-MEL-1, ZR-75-1, T-47D, Hep-G2, Hs578T, Caco-2, SK-BR-3, SK-OV-3, HCT116, MDA-MB-468, SW480, PC-3, HGC27, MDA-MB-231, HT29, Caki-1, TF-1, MCF-7, PANC1, HuH-6, A431, MGC80-3, SGC-7901, MDA-MB-453, 769-P, ACHN, OSRC-2, and SK-NEP-1) and human renal tubular epithelial (HK2) cells were purchased from the Kunming Institute of Zoology, Chinese Academy of Sciences (Kunming, Yunnan, China). The cells were cultured in DMEM high-glucose medium, DMEM/F12, or RPMI 1640 medium (HyClone, Novato, CA, United States) supplemented with 10% fetal bovine serum (FBS) (BI, Kibbutz Beit-Haemek, Israel) and 1% penicillin–streptomycin (Solarbio, Beijing, China) and cultured in a cell incubator at 37°C and with 5% CO_2_.

### MTT Assay

Cells from 30 cancer cell lines were separately seeded in 96-well plates at a density of 1 × 10^4^ cells/well. Once the cells had adhered, they were treated with 10 μM MIC-1 for 48 h, with untreated cells serving as the control. After 48 h, 20 μl of 50 mg/ml MTT reagent was added to each well. After incubating for 4 h in the dark, the supernatant was removed, 200 μl of DMSO was added to each well, followed by shaking for 10 min. The absorbance of each well was measured at 492 nm using a microplate reader (Molecular Devices Co., CA, United States).

Cells of six renal cancer cell lines (786-O, 769-P, Caki-1, ACHN, OSRC-2, SK-NEP-1) were separately seeded in 96-well plates at a density of 1 × 10^4^ cells/well. Once they had adhered, the cells were treated with MIC-1 (0, 1, or 10 µM) for 48 h. Cell viability was then evaluated using the MTT assay.

The 786-O, 769-P, and HK2 cell lines (1 × 10^4^ cells/well) were separately seeded in 96-well plates for 24 h and treated with MIC-1 (0, 1, 2, 4, 8, or 16 µM) for 24 h or 48 h. Cell viability was then evaluated using the MTT assay.

Untransfected and PTP1B-overexpressing 786-O cells (1 × 10^4^ cells/well) were separately seeded in 96-well plates for 24 h and treated with MIC-1 (0, 2, or 4 μM) for 48 h. Cell viability was then evaluated using the MTT assay.

### Colony Formation Assay

The cell lines 786-O and 769-P were separately seeded in 6-well plates at a density of 3 × 10^3^ cells/well. Once they had adhered, the cells were treated with MIC-1 (0, 1, 2, and 4 μM) for 24 h or 48 h. After 24 or 48 h, the supernatant was removed, 3 ml of normal medium was added to each well, and then the cells were incubated for 12 days. The medium was changed every 2 days. After 12 days, the supernatant was removed, after which the cells were washed twice with PBS, fixed in methanol for 10 min, stained with 0.1% crystal violet (1 ml) for 15–30 min, washed with distilled water, air-dried, and imaged. A total of 600 µl of 10% glacial acetic acid was added and mixed well. The absorbance was measured at 562 nm using a microplate reader.

Untransfected and PTP1B-overexpressing 786-O cells (3 × 10^3^ cells/well) were separately seeded in 6-well plates for 24 h and treated with MIC-1 (0, 2, or 4 μM) for 48 h. Colony numbers were then evaluated as described above.

### Wound Healing Assay

The cell lines 786-O and 769-P were separately seeded in 6-well plates at a density of 1 × 10^5^ cells/well. Once the cells had adhered, a sterile micropipette tip was used to make a scratch in the cell monolayer. After removing the supernatant, the cells were treated with different concentrations of MIC-1 (0, 1, or 2 μM), and imaged at 0 and 24 h under an inverted microscope to observe and record wound healing. The cell migration rate was then measured and analyzed using ImageJ software and GraphPad Prism 5.0 software (San Diego, CA, United States). The same procedure was followed to compare differences in cell migration between untransfected and PTP1B-overexpressing 786-O cells.

Untransfected and PTP1B-overexpressing 786-O cells (1 × 10^5^ cells/well) were seeded separately in 6-well plates for 24 h and treated with MIC-1 (0, 1, or 2 μM) for 24 h. The cell migration rate was then evaluated as described above.

### Cell Migration Assay

A serum-free 786-O and 769-P cell suspension (5 × 10^5^ cells/ml; 100 µl) containing different concentrations of MIC-1 (0, 1, or 2 µM) was separately added to the upper chamber of a Transwell insert. Then, 600 µl of medium containing 20% FBS was added to the lower chamber of the Transwell. After 24 h of incubation, the supernatant was discarded, the cells were washed twice with precooled PBS, fixed in methanol for 20 min, and finally stained with 0.1% crystal violet for 30 min. The unmigrated cells in the upper layer of the chamber were gently cleared with a cotton swab. After washing three times with precooled PBS, the migrated cells were photographed under an inverted microscope in five randomly selected fields. The number of cells in each image was counted using ImageJ software. The data are presented as percentage of cell migration relative to control.

### Cell Invasion Assay

Matrigel and serum-free culture medium were mixed in a 1:6 ratio. Then, 100 µl of the mixture was added to the upper chamber of a Transwell insert and incubated for 24 h. After washing the Matrigel with serum-free medium, 100 µl of a serum-free 786-O and 769-P cell suspension (5 × 10^5^ cells/ml) containing different concentrations of MIC-1 (0, 1, or 2 µM) was separately added to the upper chamber of the transwell insert, while 600 µl of medium containing 20% FBS was added to the lower chamber. After 24 h of incubation, the supernatant was removed, the cells were washed twice with precooled PBS, fixed in methanol for 20 min, and stained with 0.1% crystal violet for 30 min. Unmigrated cells in the upper layer of the chamber were gently washed off with a cotton swab, while the invading cells were photographed under an inverted microscope in five randomly selected fields. The number of cells in each image was counted using ImageJ software. The data are presented as percentage of cell migration relative to control.

### Flow Cytometric Analysis

The cell lines 786-O and 769-P in the logarithmic growth phase were seeded in a 6-well plate at 2 × 10^5^ cells/well. Once they had adhered, the cells were treated with MIC-1 (0, 1, 2, or 4 μM) for 48 h. The cells were then collected, washed three times with precooled PBS, centrifuged, and the supernatant removed. The cells were resuspended in 100 µl of binding buffer containing Annexin V-FITC and propidium iodide (PI), mixed gently, and incubated for 15 min at room temperature in the dark. The cell signals were detected using a flow cytometer (BD, FACSCalibur, CA, United States), and the apoptosis rate was assessed by FlowJo software (Treestar, United States).

To determine the cell cycle distribution, cells were seeded and treated as above. After 48 h, the cells were collected and fixed in 70% ethanol at 4°C overnight, centrifuged, the supernatant discarded, the cells were washed again with precooled PBS, and then resuspended in 400 µl of PI staining solution containing RNase. After incubating at 37°C for 30 min in the dark, the cells were analyzed using a flow cytometer, and the cell cycle distribution was analyzed using FlowJo software (Treestar).

### Western Blotting Analysis

The cell lines 786-O, 769-P, HCT-116, Hep-G2, and A431 (1 × 10^6^) in the logarithmic growth phase were seeded in a 5-cm dish. Once they had adhered, the cells were treated with MIC-1 (0, 2, or 4 μM) for 48 h. Total cellular protein was then extracted with RIPA lysis buffer (Solarbio) and quantified by the BCA method. Equal amounts of protein were separated by 8% or 10% SDS-PAGE, transferred to polyvinylidene fluoride (PVDF) membranes (Millipore, Billerica, MA, United States), blocked with 5% bovine serum albumin (BSA) (Solarbio) for 30 min, and then incubated overnight at 4°C with primary antibodies targeting Bax, Bcl-2, CDK2, K-Ras, and cyclinD1 (Cat. #sc-7480, sc-7382, sc-6248, sc-30, and sc-8396, respectively; 1:1,000 dilution; Santa Cruz Biotechnology, CA, United States), PTP1B and p-Src (Tyr529) (Cat. #ab244207, 32,078; 1:2000 dilution; Abcam, Cambridge, MA, United States), Src, p-Src (Tyr416), cyclinA2, Raf, p-Raf, ERK1/2, p-ERK1/2, and *β*-actin (Cat. #2109, 59,548, 4,656, 9422S, 9,431, 4,695, 4,370, and 3,700, respectively; 1:2000 dilution; Cell Signaling Technology, Danvers, MA, United States). The following day, the membranes were incubated for 1 h with horseradish peroxidase (HRP)-conjugated secondary IgG antibody (1:10,000, R&D Systems, United States). Signals were detected using the ECL Western blotting detection system (FluorChem E Protein Simple).

Untransfected and PTP1B-overexpressing 786-O cells were processed as above to detect protein expression levels.

The tumor tissue protein was extracted and the concentration was determined. Protein expression levels were determined as described above.

### Enzyme Linked Immunosorbent Assay

The cell lines 786-O and 769-P in the logarithmic growth phase were seeded in a 6-well plate at 2 × 10^5^ cells/well. Once they had adhered, the cells were treated with MIC-1 (0, 1, or 2 μM) for 24 h. The amount of matrix metalloproteinase (MMP)-2 and MMP-9 released from cell culture supernatants were measured by an ELISA kit (Mei Mian Biotechnology Co., Ltd., Jiangsu China) according to the kit instructions.

### Target Prediction Using PharmMapper

The SDF file of the MIC-1 molecular structure was downloaded from the PubChem database, and uploaded to the PharmMapper server (http://59.78.96.61/pharmmapper). The search was started by selecting a library of 2,241 human disease-related target proteins as the screening object, selecting the allowable conformational change parameters, and setting the maximum number of conformations for each molecule to 100. Target proteins were ranked according to the comprehensive match value Z′-score.

### PTP1B Inhibition Assay

The *in vitro* inhibitory activity of MIC-1 against PTP1B was determined using a PTP1B Inhibitor Screening Assay Kit (ab139465, Abcam). In brief, a phosphate standard curve was generated by adding different volumes of PTP1B assay buffer (100, 97.5, 95, 90, 80, and 70 μl) and different volumes of 100 μM phosphate standard solutions (0, 2.5, 5, 10, 20, and 30 μl) to each well. Next, 10 μl of different concentrations of MIC-1 (10, 20, 40, 80, 160, or 320 μM) or the positive control suramin (1, 5, 10, 20, 40, or 80 μM) were mixed with 35 μl of PTP1B assay buffer and 5 μl of PTP1B diluent, giving a total reaction volume of 50 μl. A “time zero” (10 μl of dimethyl sulfoxide [DMSO], 35 μl of PTP1B assay buffer, and 5 μl of PTP1B diluent) and a “control well” (10 μl of DMSO and 40 μl of PTP1B assay buffer) were also prepared. Next, 50 μl of PTP1B substrate was added to each well and incubated at 37°C for 30 min. Then, 25 μl of red assay reagent was added to stop the reaction, and, after 20 min, absorbance was measured at 620 nm using a microplate reader. The optical density (OD) value was converted to nmol PO_4_^2−^ using a standard curve. Activity was calculated as the percent (%) of Control: % Activity = [Test sample (nmol PO_4_^2−^)—“time zero” (nmol PO_4_^2−^)] [Control (nmol PO_4_^2−^)—“time zero” (nmol PO_4_^2−^)] × 100.

### TC-PTP Inhibition Assay

Aliquots (10 μl) of different concentrations of MIC-1 (10, 20, 40, 80, 160, or 320 μM) were mixed with 50 μl of TC-PTP (40 μg/ml), and the mixture was incubated at room temperature for 10 min. Next, 50 μl of 1 mM *para*-nitrophenyl phosphate (*p*NPP) was added to the mixture, followed by incubation at 37°C for 30 min. Finally, 50 μl of NaOH (3 M) was added to stop the reaction. The absorbance was measured at 405 nm using a microplate reader.

### Surface Plasmon Resonance Studies

The binding affinity of MIC-1 and PTP1B (Sino Biological, Cat. 10,304-H07E) was determined with a Biacore S200 system (GE Healthcare, Uppsala, Sweden). PTP1B was immobilized on the Fc2 channel of the CM5 chip using an amine-coupling kit (GE Healthcare) following the manufacturer’s protocol. PTP1B was diluted to 100 μg/ml with a 10 mM sodium acetate solution (pH 4.0). Kinetics and affinity were determined at a flow rate of 30 μl/min using PBS-P buffer. MIC-1 association and dissociation rates were measured for 90 s each. The KD values were calculated using the kinetics and affinity analysis option of the Biacore S200 Evaluation Software Version 1.1 (GE Healthcare).

### Molecular Docking and Small Molecule-Protein Interaction Model Prediction

The molecular docking analysis was performed using the CDOCKER program of Discovery Studio (DS) 4.5 software (Accelrys Software Inc., San Diego, CA, United States). The X-ray crystal structure of PTP1B (PDB ID: 1NWE) was retrieved from the Protein Data Bank (PDB). The macromolecular structure of the PTP1B protein was selected as the receptor, and the protein molecules were optimized and prepared by adding hydrogen atoms, dewatering molecules, and applying the CHARMm force field. The binding site of the protein molecule was determined by the binding site of the known original ligand and the target, and the binding pocket radius range was automatically generated. The structure of MIC-1 was converted into a three-dimensional (3D) molecular formula, and the “Prepare Ligand” module was used to add hydrogen atoms and perform energy optimization operations. The parameters were set to default values. After molecular docking, the 10 best small molecule–protein binding conformations were obtained. The binding free energy and binding mode simulations (two-dimensional [2D] and 3D graphs) of the ligand-receptor interaction were also generated after the CDOCKER process.

### Transfection of PTP1B cDNA Into 786-O Cells

The PTP1B overexpression cell line was generated using the piggyBac (PB) transposon system. The cell line 786-O (4 × 10^5^ cells/well) was seeded into 6-well plates and the PTP1B cDNA vector was transfected into the cells using Lipofectamine 3,000 Reagent (Thermo Fisher Scientific, Waltham, MA, United States). After selection with 1 μg/ml puromycin, clones expressing PTP1B were established.

### RT-PCR Assay

Total RNA was extracted from PTP1B-overexpressing cells using Trizol reagent. The PCR reaction was carried out in a programmable thermal cycler. The PTP1B primer pair used was PTP1B-F (5′-GGC​CAT​TTA​CCA​GTT​GAC​CA-3′) and PTP1B-R (5′-ATG​ACG​ACA​CCC​CTG​CTT​TT-3′), which was expected to generate a 185-bp product. The GAPDH housekeeping gene primer pair GAPDH-F (5′-GCT​CTC​TGC​TCC​TCC​TGT​TC-3′) and GAPDH-R (5′-TTC​CCG​TTC​TCA​GCC​TTG​AC-3′) was expected to generate a 273-bp product.

### Xenograft Model in Nude Mice

Twenty-four 6-week-old male BALB/C nude mice were purchased from Changzhou Cavans Laboratory Animal Co., Ltd. After 1 week of adaptive feeding, 786-O cells (5 × 10^6^) were subcutaneously injected into each mouse. Once the tumor volume had reached 100 mm^3^, the mice were randomly divided into four groups, including a control group, a sunitinib (25 mg/kg) group, a MIC-1 low-dose (25 mg/kg) group, and a MIC-1 high-dose (50 mg/kg) group. Gavage was carried out once every 2 days, and the tumor volume, body weight, food intake, and water intake were recorded. After 3 weeks, the mice were euthanized with CO_2_, and the tumor tissues were excised and stored at −80°C until use. The animal experiment protocols were reviewed and approved by the Institutional Animal Ethical Committee of Yunnan Agricultural University (IACUC-20190824-12).

### Statistical Analysis

Data presented as bar graphs are the means ± standard error of the mean (SEM) of at least three independent experiments. Statistical significance was evaluated by Student’s t-tests, one-way ANOVA, or two-way ANOVA using GraphPad Prism 5.0. *p <* 0.05, *p <* 0.01, or *p <* 0.001 was considered statistically significant.

## Results

### MIC-1 Inhibited the Growth of RCC Cells

To determine the anticancer activity of MIC-1, cells from 30 cancer cell lines were treated with MIC-1 (0 or 10 μM) for 48 h. The results showed that MIC-1 significantly inhibited the growth of 26 of the 30 cancer cell lines (LoVo, PC-12, A375, 786-O, SK-Hep-1, SH-SY5Y, SK-MEL-1, Hep-G2, Hs578T, Caco-2, SK-BR-3, SK-OV-3, HCT116, MDA-MB-468, SW480, MDA-MB-453, ZR-75-1, T-47D, HGC27, MGC80-3, MCF-7, PANC1, MDA-MB-231, HT29, PC-3, and Caki-1), but had no effect on the growth of the other four cell lines (TF-1, HuH-6, A431, and SGC-7901). MIC-1 exerted the strongest growth inhibitory effects on 786-O RCC cells, with the growth inhibitory rate reaching 88.69 ± 2.26% (Figure 1B).

To further investigate the effects of MIC-1 on the growth of different renal cancer cells, 786-O, 769-P, Caki-1, ACHN, OSRC-2, and SK-NEP-1 cells were treated with MIC-1 (0, 1, or 10 μM) for 48 h. Compared with the control, treatments with low doses of MIC-1 significantly inhibited the growth of 786-O cells (growth inhibition rate = 36.3 ± 9.3%) and SK-NEP-1 cells (growth inhibition rate = 8.52 ± 1.34%), but not the growth of the other four renal cancer cells. Meanwhile, high-dose MIC-1 treatment significantly inhibited the growth of ACHN, SK-NEP-1, 769-P, OSRC-2, and 786-O cells, with growth inhibition rates of 35.92 ± 3.79%, 51.01 ± 2.99%, 59.99 ± 7.38%, 69.46 ± 13.23%, and 85.57 ± 2.11%, respectively (Figure 1C). These results indicated that MIC-1 can inhibit the growth of RCC cells.

To investigate whether the growth inhibitory effects of MIC-1 on 786-O and 769-P RCC cells were dose- and time-dependent, we treated 786-O and 769-P cells with different concentrations of MIC-1 (0, 1, 2, 4, 8, or 16 μM) for 24 or 48 h. The results showed that MIC-1 treatment decreased the viability of 786-O and 769-P cells in a dose- and time-dependent manner (Figures 1D,E).

The colony formation assay results indicated that MIC-1 (0, 1, 2, and 4 μM) significantly decreased the colony-forming ability of 786-O and 769-P cells compared with that of the control group. At 24 h, the colony-formation rate of 786-O cells decreased from 100.00 ± 1.66% to 87.40 ± 1.71%, 27.50 ± 0.71%, and 22.95 ± 0.22%, respectively; At 48 h, the colony-formation rate of 786-O cells decreased from 100.00 ± 3.61% to 69.44 ± 2.80%, 26.58 ± 0.44%, and 18.34 ± 0.15%, respectively (Figure 1F). Meanwhile, at 24 h, the colony-formation rate of 769-P cells decreased from 100.00 ± 6.07% to 87.73 ± 8.58%, 57.19 ± 4.70%, and 18.99 ± 2.04%, respectively; At 48 h, the colony-formation rate of 769-P cells decreased from 100.00 ± 3.58% to 70.84 ± 2.42%, 51.13 ± 4.56%, and 11.94 ± 0.55%, respectively (Figure 1G).

To determine whether MIC-1 was toxic to normal renal cells, we evaluated the effects of MIC-1 on the growth of renal tubular epithelial cells (HK2 cells) using the MTT assay. Even when the dose was as high as 8 μM, MIC-1 did not negatively influence the viability of HK2 cells (Figure 1H). These results indicated that MIC-1 could significantly inhibit the growth of RCC cells, but was not toxic to normal renal cells.

### MIC-1 Inhibited the Migratory and Invasive Ability of RCC Cells

**FIGURE 2:**
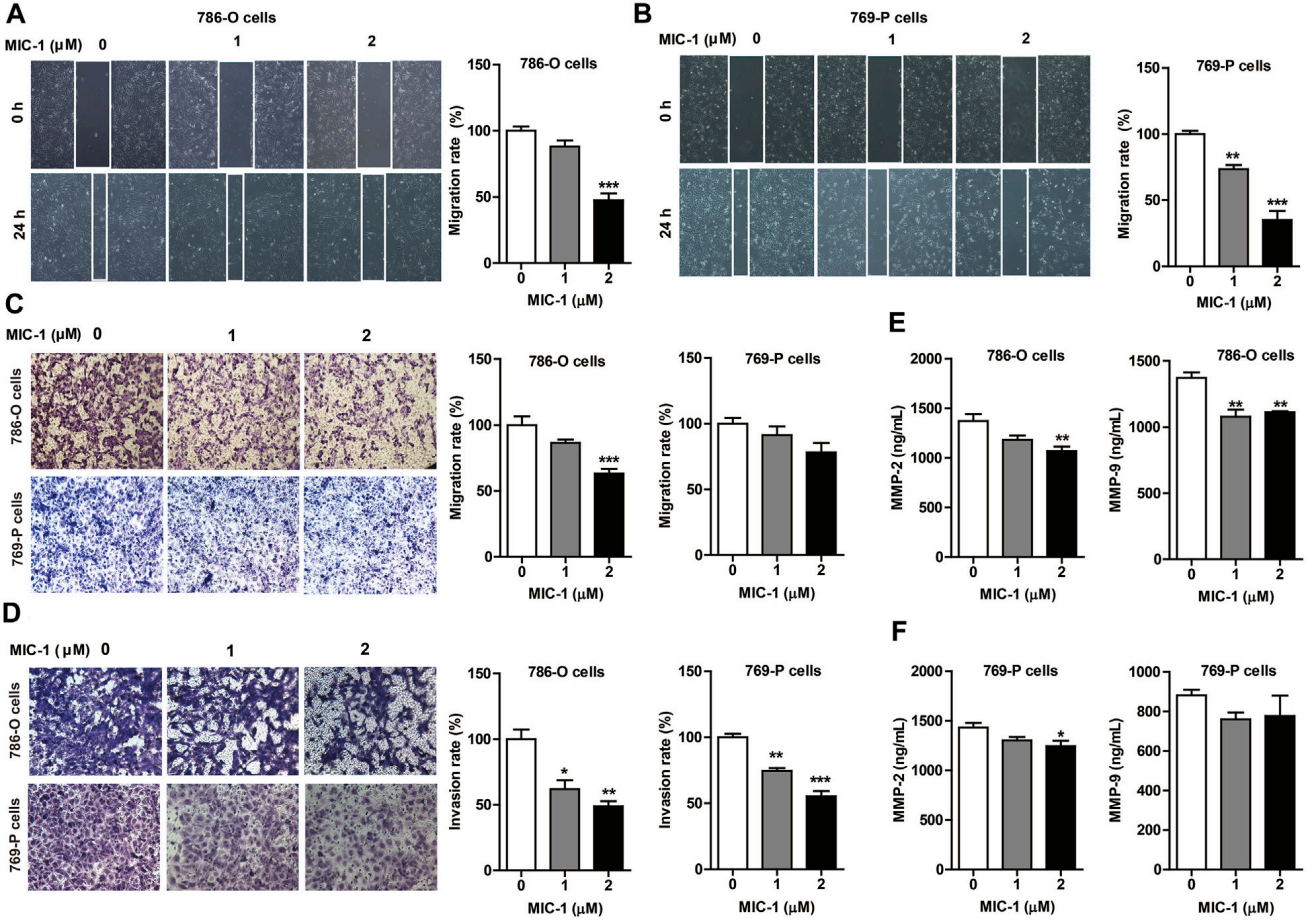
MIC-1 inhibits the migratory and invasive capability of RCC cells. 786-O cells and 769-P cells were treated with MIC-1 (0, 1, or 2 μM) for 24 h. The migratory ability of 786-O cells and 769-P cells were analyzed using a wound healing assay **(A,B)** and a Transwell assay **(C)**, while their invasive ability were analyzed using a Transwell assay with Matrigel-coated inserts **(D)**. Migrated or invaded cells were counted in five randomly selected fields. The number of cells in each image was counted using the ImageJ software. The data are presented as percentage of cell migration or invasion relative to control. **(E,F)** The expression of MMP-2 and MMP-9 in supernatant of 786-O cells and 769-P cells was detected by double-antibody one-step sandwich enzyme-linked immunosorbent assay. Values represent the means ± SEM from three independent experiments and statistical analysis was performed by one-way ANOVA; *p < 0.05, **p < 0.01, ***p < 0.001: MIC-1 compared with the control (0 μM).

As most patients with advanced RCC experience distant metastases (Yu et al., 2020), we assessed the effects of MIC-1 on the migration and invasion of 786-O and 769-P cells. The results showed that exposure to MIC-1 (0, 1, and 2 μM) significantly inhibited the migration of 786-O and 769-P cells. The cell migration rate of 786-O cells decreased from 100.00 ± 3.17% to 88.00 ± 4.63% and 47.35 ± 5.31% in the wound healing assay, and from 100.00 ± 6.44% to 86.47 ± 2.49% and 63.08 ± 3.67% in the Transwell assay (Figures 2A,C). The cell migration rate of 769-P cells decreased from 100.00 ± 1.85% to 72.49 ± 3.00% and 34.73 ± 7.05% in the wound healing assay, and from 100.00 ± 4.38% to 91.39 ± 6.44% and 78.15 ± 7.19% in the Transwell assay (Figures 2B,C). In addition, compared with the control cells, MIC-1 (0, 1 and 2 μM) significantly inhibited the invasive ability of 786-O and 769-P cells. The invasion rate of 786-O cells decreased from 100.00 ± 7.21% to 61.92 ± 6.82% and 48.95 ± 3.79% (Figure 2D); the invasion rate of 769-P cells was decreased from 100.00 ± 2.62% to 74.55 ± 2.18% and 55.31 ± 3.78% (Figure 2D).

Metastasis could be simplified into two phases: translocation to a distant tissue and colonization. The initial step requires the cell with the ability to degrade and move through extracellular matrix of the surrounding tissues. Among the MMPs family, MMP2 and MMP9 are involved in the breakdown of extracellular matrix in normal processes, so they are closely associated with the cancer cell migration. To further clarify the effects of MIC-1 on the migration of 786-O and 769-P cells, we detected the expression of MMP-2 and MMP-9 by ELISA. The results showed that the expression of MMP-2 and MMP-9 released from the cells treated with MIC-1 was markedly decreased compared to control cells (Figures 2E,F). These results indicated that MIC-1 inhibited the migratory and invasive of RCC cells by suppressing the expression of MMP-2 and MMP-9.

### MIC-1 Induced Apoptosis and Cell Cycle Arrest in RCC Cells

**FIGURE 3:**
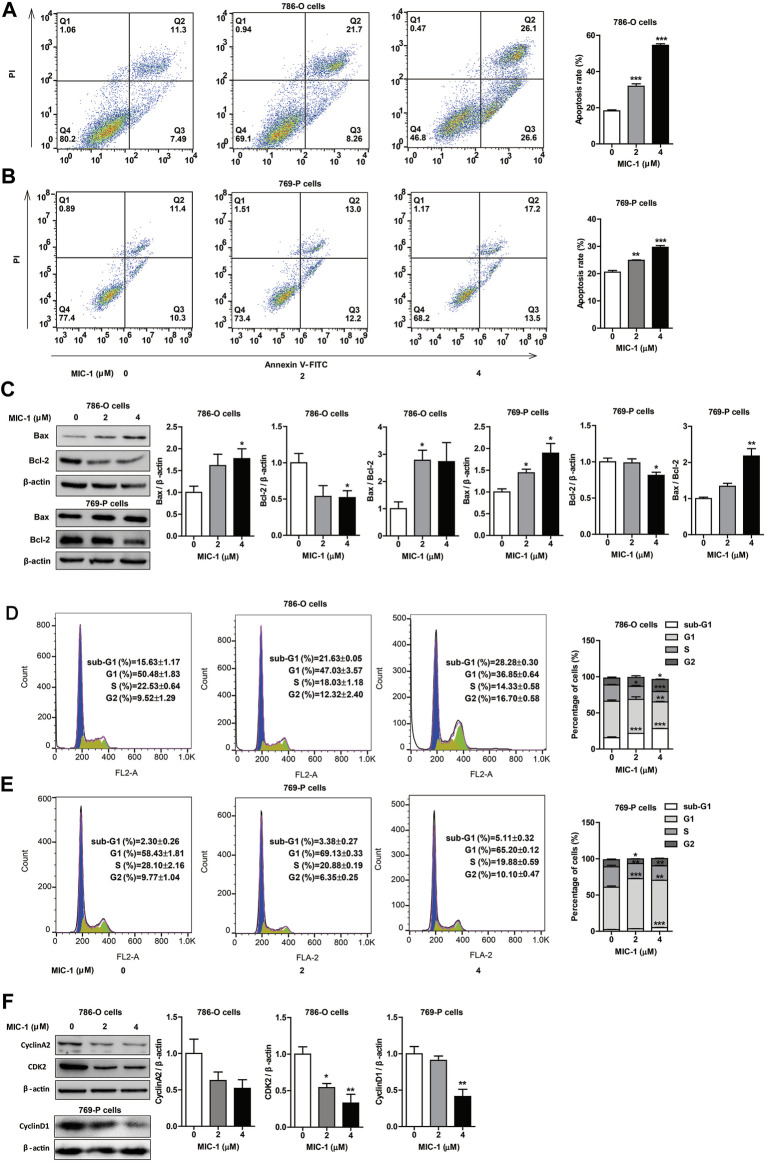
MIC-1 induces apoptosis and cell cycle arrest in RCC cells. 786-O cells and 769-P cells were treated with MIC-1 (0, 2, or 4 μM) for 48 h **(A,B)** The cell apoptosis of 786-O cells and 769-P cells were determined by flow cytometry. **(C)** The protein expression of Bax and Bcl-2 in 786-O cells and 769-P cells were detected by western blotting. *β*-actin served as a loading control. **(D,E)** The cell cycle distribution of 786-O cells and 769-P cells were determined by flow cytometry. **(F)** The protein expression of CDK2 and cyclinA2 in 786-O cells, and the protein expression of cyclinD1 in 769-P cells were detected by western blotting. *β*-actin served as a loading control. Values represent the means ± SEM from three independent experiments and statistical analysis was performed by one-way ANOVA; *p < 0.05, **p < 0.01, ***p < 0.001: MIC-1 compared with the control (0 μM).

To determine whether the inhibitory effect of MIC-1 on RCC cell growth was associated with apoptosis and cell cycle arrest, we used flow cytometry to evaluate the apoptosis rate and cell cycle distribution of 786-O and 769-P cells following treatment with different doses (0, 2, or 4 µM) of MIC-1 for 48 h. The results showed that MIC-1 induced the apoptosis of 786-O and 769-P cells in a dose-dependent manner. Compared with the control group (18.24 ± 0.55%), the apoptotic rate of 786-O cells treated with MIC-1 increased to 31.85 ± 1.33% and 54.44 ± 0.83%, respectively (Figure 3A), while the apoptotic rate of 769-P cells treated with MIC-1 increased from 19.10 ± 0.20% to 24.95 ± 0.52% and 29.42 ± 0.86%, respectively (Figure 3B). In addition, MIC-1 significantly increased the protein expression of the proapoptotic proteins Bax, decreased the expression of the antiapoptotic protein Bcl-2, and increased the Bax/Bcl-2 ratio in 786-O cells and 769-P cells (Figure 3C).

Furthermore, after MIC-1 treatment, the percentage of 786-O cells in the G2 phase of the cell cycle increased from 5.42 ± 1.52% in the control cells to 12.54 ± 1.79% and 25.32 ± 0.45%, respectively, while the percentage of cells in the S-phase decreased from 21.55 ± 1.58% in the control cells to 15.46 ± 0.67% and 6.87 ± 0.84%, respectively (Figure 3D). The percentage of 769-P cells in the G1 phase of the cell cycle increased from 58.43 ± 1.81% in the control cells to 69.13 ± 0.33% and 65.20 ± 0.12%, respectively (Figure 3E). We further determined the expression levels of cell cycle-related proteins in 786-O cells using western blotting, and found that MIC-1 downregulated the expression level of CDK2 and cyclinA2. In addition, MIC-1 significantly downregulated the expression level of cyclinD1 in 769-P cells (Figure 3F). These results suggested that MIC-1 inhibits the proliferation of RCC cells by inducing apoptosis and cell cycle arrest.

### MIC-1 Selectively Inhibited PTP1B Activity

Computer-aided drug screening is a fast and accurate method to predict drug-protein interactions. To investigate the molecular mechanism underlying the MIC-1-related inhibitory effects on the growth and migration of 786-O cells, we used the PharmMapper network database to conduct reverse virtual screening of MIC-1 target proteins. Among the 10 target proteins with the highest Z′-score filtered from the target library of 2,241 human disease-related proteins—platelet glycoprotein Ib alpha chain (GPIb-alpha), histo-blood group ABO system transferase, CD209 antigen, peptidyl-prolyl *cis*-*trans* isomerase (PPIase) FKBP1B, PTP1B, glucosamine-6-phosphate isomerase (GNPDA), phenylalanine-4-hydroxylase (PAH), proto-oncogene serine/threonine-protein kinase pim-1, beta-hexosaminidase subunit beta (HEXB), and sorbitol dehydrogenase (SDH) (Supplementary Table S1), PTP1B was found to be the most closely related to cancer occurrence and development (Bollu et al., 2017).

**FIGURE 4:**
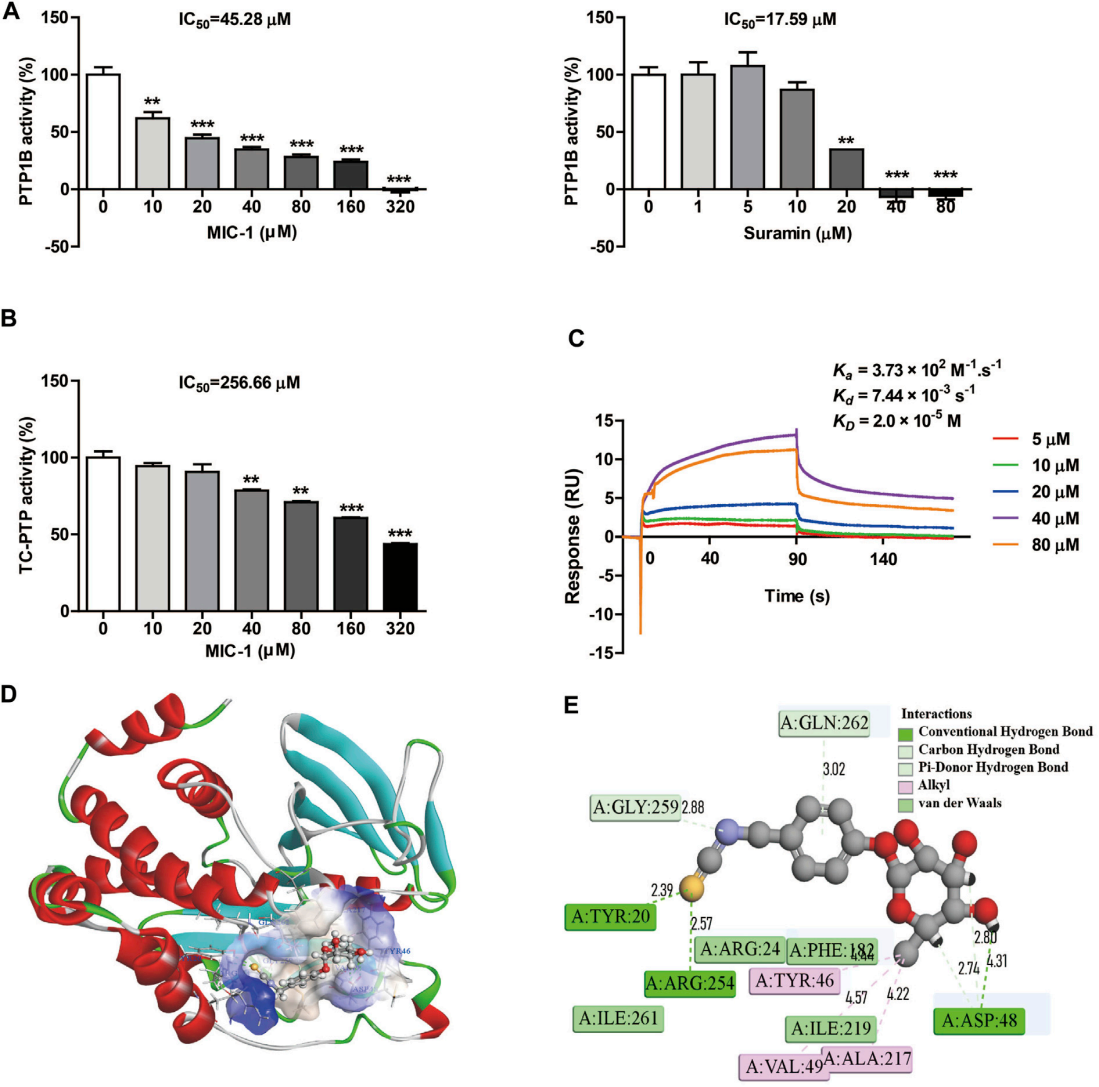
MIC-1 selectively inhibits PTP1B activity. **(A)** The inhibitory activities of MIC-1 and suramin against PTP1B. **(B)** The inhibitory activity of MIC-1 against TC-PTP. **(C)** MIC-1 directly binds to PTP1B with high affinity (KD = 2.0 × 10^−5^ M). Biolayer interferometry (BLI) sensorgrams indicating the interactions between gradient concentrations of MIC-1 and PTP1B were measured on an Octet Red96 system, with association and dissociation for 90 s each. **(D)** Three-dimensional simulation of the interaction between MIC-1 and the PTP1B protein. **(E)** Two-dimensional simulation of the interaction between MIC-1 and the PTP1B protein. Values represent the means ± SEM from three independent experiments and statistical analysis was performed by one-way ANOVA; **p < 0.01, ***p < 0.001: MIC-1 compared with the control (0 μM).

To investigate the effects of MIC-1 on the activity of PTP1B, an *in vitro* enzyme activity assay was carried out. The results showed that MIC-1 significantly inhibited the activity of PTP1B in a dose-dependent manner, with an IC_50_ of 45.28 μM. The IC_50_ of the positive control suramin was 17.59 μM (Figure 4A). T-cell protein tyrosine phosphatase (TC-PTP) shared the greatest sequence similarity with PTP1B (74% identity) in the catalytic domain and had identical active sites. Therefore, to verify that MIC-1 exerted selective inhibitory effect on PTP1B, we tested the enzyme activity of TC-PTP under MIC-1 treatment. We found that MIC-1 exerted only weak inhibitory activity against TC-PTP (IC_50_ = 256.66 μM) (Figure 4B).

Next, we performed a binding assay to determine whether MIC-1 could directly bind to PTP1B. The results showed that PTP1B directly interacted with MIC-1 and that MIC-1 had a relatively high affinity for PTP1B, with an equilibrium dissociation constant (KD) of 2.0 × 10^−5^, an association rate constant (*k*_on_) (1/ms) of 3.73×10^2^, and a dissociation rate constant (*k*_off_) (1/s) of 7.44 × 10^−3^ (Figure 4C).

To predict the molecular binding mode of MIC-1 to the PTP1B protein, we used the molecular docking program CDDDock. Molecular docking analysis showed that MIC-1 and PTP1B have a high binding capacity, with a molecule-protein binding energy of −50.0513 kcal/mol. Analysis of the specific binding mode indicated that MIC-1 fit well into the hydrophobic pocket of the active site of the PTP1B protein (Figure 4D). In addition, clear molecular interaction patterns were found in the 2D simulation diagram of the interaction between MIC-1 and PTP1B (Figure 4E). The results showed that seven hydrogen bonds and seven hydrophobic interactions could be formed between MIC-1 and PTP1B. The sulfur atom in the MIC-1 isothiocyanate functional group formed a hydrogen bond with the Tyr20 and Arg254 residues of PTP1B, while the nitrogen atom in the isothiocyanate group also formed a C hydrogen bond with the Gly259 residue of PTP1B. The aromatic ring and glycosyl side chain of MIC-1 can formed three sets of pi hydrogen bonds with the Gln262 and Asp48 residues of PTP1B. The H of the MIC-1 glycosyl side chain formed a hydrogen bond with the Asp48 residue of PTP1B, and the methyl group of the glycosyl tail formed three sets of alkyl–alkyl interactions with Tyr46, Val49, and Ala217 of PTP1B. In addition, MIC-1 also formed Van der Waals interactions with the Arg24, Phe182, Ile261, and Ile2194 residues of PTP1B. These results indicated that MIC-1 could serve as a selective inhibitor of PTP1B.

### MIC-1 Inhibited the Growth and Migration of RCC Cells Through Regulation the PTP1B-dependent Src/Ras/Raf/ERK Signaling

**FIGURE 5:**
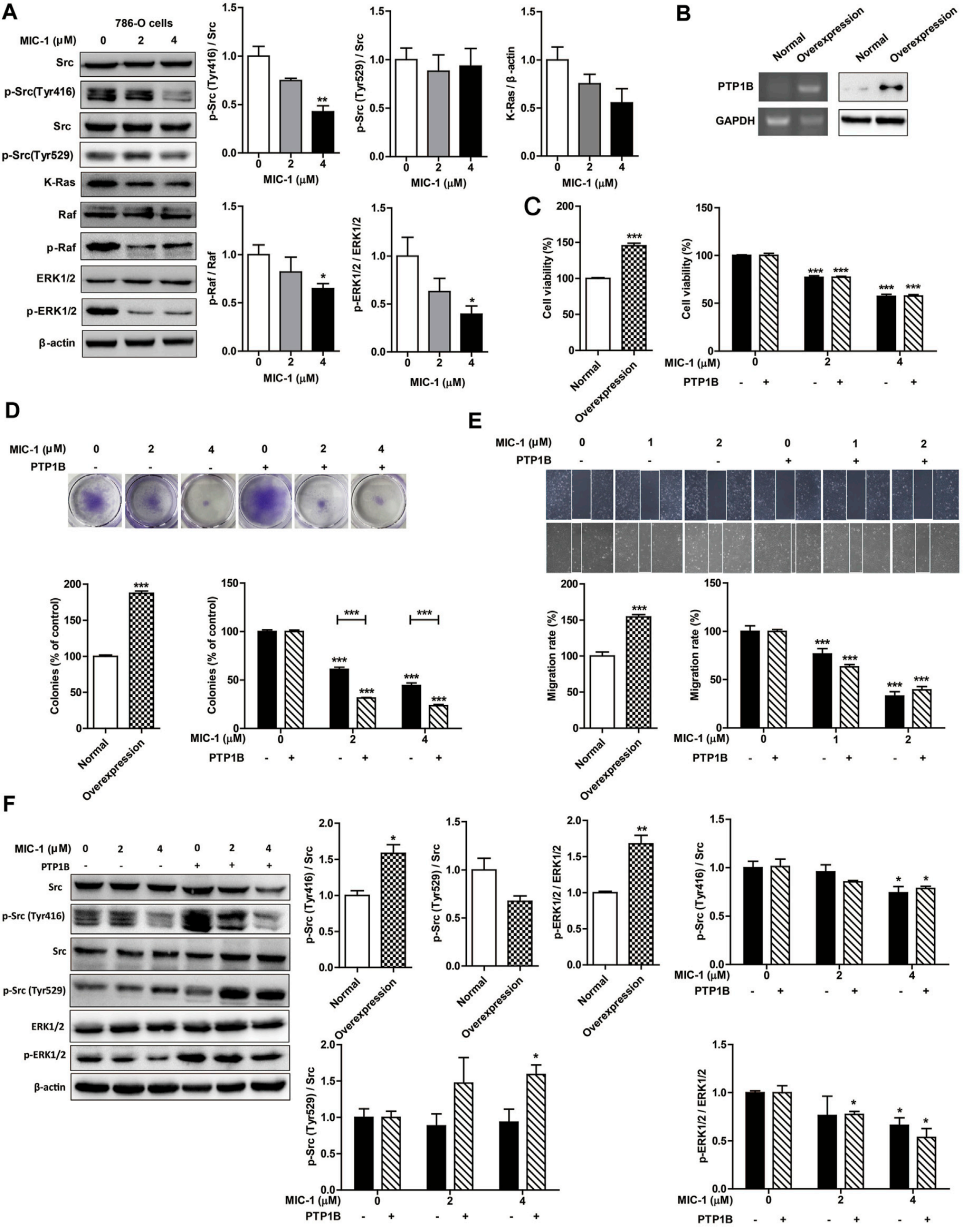
MIC-1 inhibits the growth and migration of 786-O cells by inhibiting the PTP1B-mediated activation of the Src/Ras/Raf/ERK signaling pathway. **(A)** 786-O cells were treated with MIC-1 (0, 2, or 4 μM) for 48 h, and the expression of Src, p-Src (Tyr416), p-Src (Tyr529), K-Ras, Raf, p-Raf, ERK1/2, and p-ERK1/2 was detected by western blotting. *β*-actin served as a loading control. **(B)** Detection of PTP1B mRNA and protein expression in 786-O cells transfected with a PTP1B cDNA expression vector by RT-PCR and western blotting, respectively. **(C)** Untransfected and PTP1B-overexpressing 786-O cells were treated with different concentrations of MIC-1 (0, 2, or 4 μM) for 48 h after which cell viability was analyzed by the MTT assay. **(D)** Clonogenic ability was analyzed by a colony formation assay. **(E)** Untransfected and PTP1B-overexpressing 786-O cells were treated with different concentrations of MIC-1 (0, 1, or 2 μM) for 24 h after which migratory ability was analyzed using a wound healing assay. **(F)** Untransfected and PTP1B-overexpressing 786-O cells were treated with MIC-1 (0, 2, or 4 μM) for 48 h, and the expression of Src, p-Src (Tyr416), p-Src (Tyr529), ERK1/2, and p-ERK1/2 was detected by western blotting. *β*-actin served as a loading control. Values represent the means ± SEM from three independent experiments and statistical analysis was performed by one-way ANOVA; *p < 0.05, **p < 0.01, ***p < 0.001: MIC-1-treated cells compared with control 786-O cells (0 μM) or PTP1B-overexpressing 786-O cells (0 μM); PTP1B-overexpressing 786-O cells compared with control 786-O cells.

Studies have shown that PTP1B promotes the proliferation of colon cancer, breast cancer, and lung cancer by phosphorylating and activating Src (Zhu et al., 2007; Arias-Romero et al., 2009; Liu et al., 2015), a tyrosine kinase containing an SH3 domain. Activated Src then further activates the downstream Ras/ERK signaling pathway, thereby promoting tumor cell growth and migration (Bollu et al., 2017). To further investigate the influence of MIC-1 on the PTP1B-dependent Src/Ras/Raf/ERK signaling pathway, we analyzed the expression of proteins involved in this pathway. Compared with untreated control cells, MIC-1 decreased the expression level of p-Src (Tyr416), K-Ras, p-Raf, and p-ERK1/2 (Figure 5A). These results indicated that MIC-1 blocked Src activation by inhibiting the activity of PTP1B, thereby suppressing the activation of the downstream Ras/Raf/ERK signaling pathway.

To further confirm that MIC-1 inhibits the proliferation and migration of 786-O cells by regulating the PTP1B-dependent Src/Ras/Raf/ERK signaling pathway, we established cell lines overexpressing PTP1B. We found that the mRNA and protein expression levels of PTP1B, cell proliferation rate, cell migration rate, p-Src (Tyr416) and p-ERK1/2 expression levels in 786-O cells overexpressing PTP1B were significantly higher than that in normal 786-O cells. In addition, the expression level of p-Src (Tyr529) in 786-O cells overexpressing PTP1B was lower than that in normal 786-O cells (Figures 5B–F). However, compared with untreated 786-O cells overexpressing PTP1B, MIC-1 significantly decreased the cell proliferation rate, cell migration rate, the expression of p-Src (Tyr416) and p-ERK1/2, and increased the expression of p-Src (Tyr529) (Figures 5C–F). These results indicated that MIC-1 inhibited the growth and migration of RCC cells through the PTP1B/Src/Ras/Raf/ERK signaling pathway.

### MIC-1 Inhibited the Growth of Tumors Derived From 786-O Cell Xenografts

**FIGURE 6:**
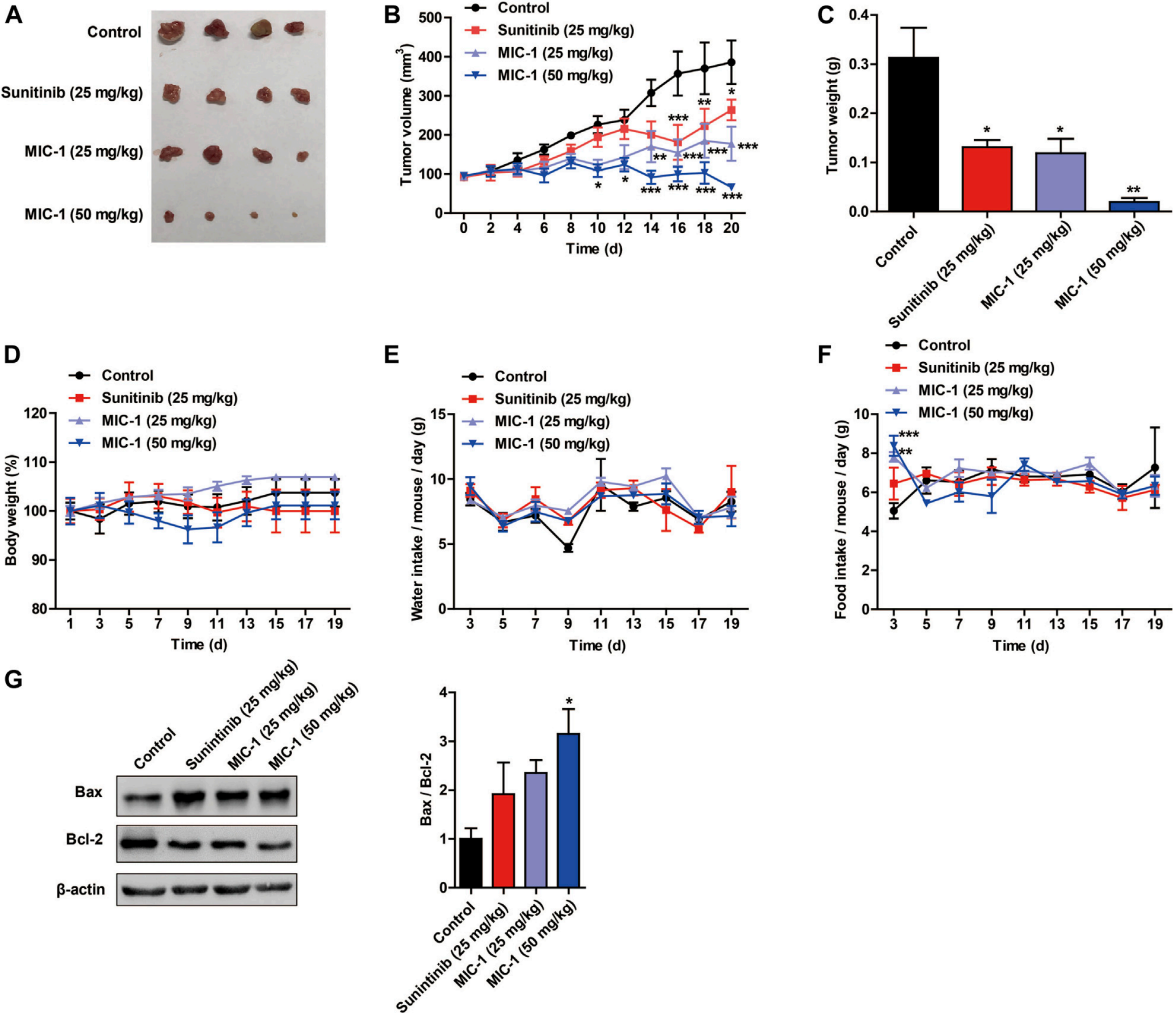
MIC-1 inhibits the growth of subcutaneous xenograft tumors *in vivo*. 786-O cells were injected subcutaneously into nude mice to evaluate the *in vivo* effects of MIC-1. **(A)** Representative images of the tumors are shown. Changes in tumor volume **(B)**, tumor weight **(C)**, body weight **(D)**, water intake **(E)**, and food intake **(F)** in the different groups of nude mice. **(G)** Western blotting analysis was used to determine the protein levels of Bax and Bcl-2, with *β*-actin serving as a loading control. The results are expressed as the means ± SEM from three independent experiments and statistical analysis was performed by one-way ANOVA or two-way ANOVA. *p < 0.05, **p < 0.01, ***p < 0.001: MIC-1 compared with the control.

To further reveal how MIC-1 inhibited the growth of 786-O cells *in vivo*, we generated a 786-O cell xenograft tumor model in nude mice. We found that, compared with the control group, MIC-1 (25 and 50 mg/kg body weight [BW]) treatment markedly suppressed the growth of 786-O cell xenograft-derived tumors in a dose-dependent manner. In addition, the inhibitory effect of low-dose MIC-1 (25 mg/kg BW) on tumor growth was equivalent to that of the positive control drug sunitinib (25 mg/kg BW) (Figures 6A–C). However, there was no significant difference in BW, water intake, and food intake between the mice of the different groups (Figures 6D–F). Next, we measured the expression levels of apoptosis-related proteins in tumor tissues. Compared with the control group, MIC-1 treatment increased the expression of Bax, decreased Bcl-2 expression, and significantly increased the Bax/Bcl-2 ratio (*p <* 0.05) (Figure 6G). These results indicated that MIC-1 could inhibit the growth of 786-O cells *in vivo*.

### Effect of MIC-1 on PTP1B-Related Src/Ras/Raf/ERK Signaling Pathway in Non-renal Cancer Cells

**FIGURE 7:**
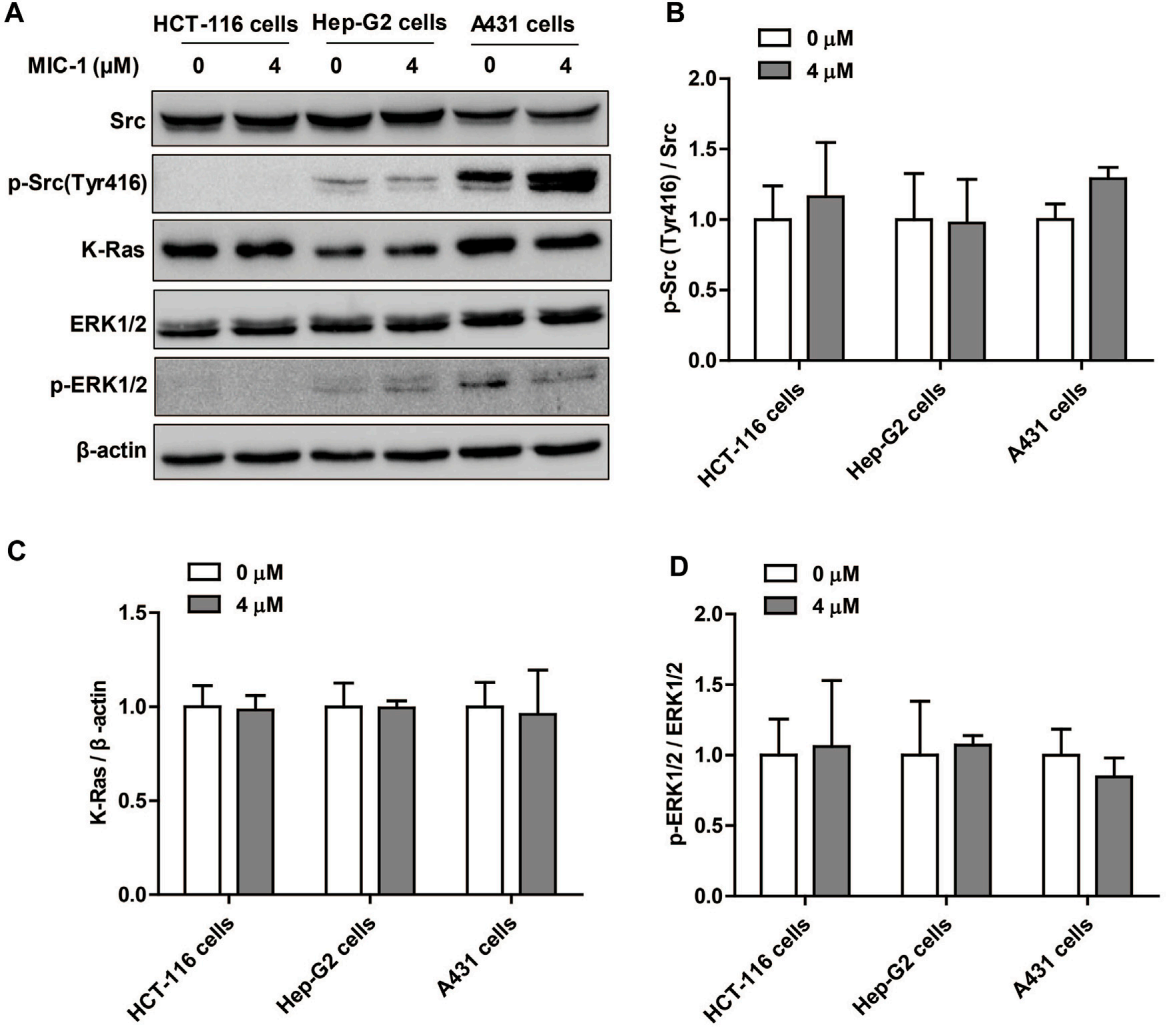
Effect of MIC-1 on PTP1B-related Src/Ras/Raf/ERK signaling pathway in non-renal cancer cells. **(A)** HCT-116 cells, Hep-G2 cells, and A431 cells were treated with MIC-1 (0 or 4 μM) for 48 h, and the expression of Src, p-Src (Tyr416), K-Ras, ERK1/2, and p-ERK1/2 was detected by western blotting. *β*-actin served as a loading control. Quantification of the relative levels of p-Src (Tyr416) **(B)**, K-Ras **(C)**, and p-ERK1/2 **(D)**; each value was normalized to that of Src, *β*-actin, and ERK1/2, respectively. Values represent the means ± SEM from three independent experiments and statistical analysis was performed by unpaired two-tailed Student’s t tests.

To investigate the regulatory effects of MIC-1 on PTP1B-related signaling pathways in non-renal cancer cells, the cancer cell lines (HCT- 116, Hep-G2, and A431) were treated with MIC-1 (0 or 4 μM) for 48 h, and then the expression of Src/Ras/Raf/ERK signaling pathway-related proteins were detected. The results showed that, compared with untreated control cells, MIC-1 exerted no effects on the expression of p-Src (Tyr416), K-Ras, and p-ERK1/2 in HCT-116 cells, Hep-G2 cells, or A431 cells (Figures 7A–D). These results indicated that MIC-1 had no effect on the PTP1B-dependent Src/Ras/Raf/ERK signaling pathway in non-renal cancer cells.

## Discussion

In traditional medicine, *M. oleifera* seeds can be used to treat many diseases (Popoola and Obembe, 2013). MIC-1 is one of the main active ingredients in *M. oleifera* seeds and has been shown to exert a variety of pharmacological activities. However, relatively few studies have reported on the anti-renal cancer effects of MIC-1. In this study, we revealed that MIC-1 has an inhibitory effect on the growth and migration of RCC cells, and that MIC-1 exerts this effect by suppressing the activation of the PTP1B/Src/Ras/ERK signaling pathway.

In both Brassicaceae and Moringaceae, when plant tissues are subjected to physical damage, such as grinding or chewing, glucosinolates are converted into ITCs under the action of endogenous myrosinase (a glucosinolate glycohydrolase) (Waterman et al., 2014; Förster et al., 2015). There are four unique sugar-modified aromatic glucosinolates in *M. oleifera* (Bennett et al., 2003). Under certain conditions, these glucosinolates can be converted *in situ* into four ITCs, specifically, MIC-1; 4-[(4′-O-acetyl-α-L-rhamnosyloxy) benzyl] isothiocyanate; 4-[(2′-O-acetyl-α-L-rhamnosyloxy) benzyl] isothiocyanate; and 4-[(3′-O-acetyl-α-L-rhamnosyloxy) benzyl] isothiocyanate (Waterman et al., 2014). MIC-1 and 4-[(4′-O-acetyl-α-L-rhamnosyloxy) benzyl] isothiocyanate are the most abundant of these ITCs, accounting for more than 95% of the total. Compared with other ITCs in cruciferous plants, such as sulforaphane, studies investigating MITCs remain relatively rare. Because MITCs retain a rhamnose sugar moiety in their molecular structure, they have greater stability and similar or stronger biological activity relative to other ITCs in cruciferous plants (Brunelli et al., 2010; Cheenpracha et al., 2010; Park et al., 2011; Tumer et al., 2015), which renders them worthy of further investigation.

MIC-1 has generated substantial interest because of its reported hypoglycemic, antioxidant, anti-inflammatory, and anticancer properties, both *in vitro* and *in vivo*. For instance, MIC-1 can inhibit the growth of different types of cancer cells by suppressing the NF-κB signaling pathway and increasing the level of glutathione-S-transferase, and was further shown to inhibit the growth of myeloma cells in CD1 mice, also by inhibiting the NF-κB pathway (Brunelli et al., 2010). MIC-1 can also induce apoptosis in human astrocytoma grade IV CCF-STTG1 cells by increasing the expression of P53 and Bax and decreasing Bcl-2 levels. Moreover, a high dose of MIC-1 can influence the expression of oxidative stress-related factors (Nrf2 and CK2α), indicating that MIC-1 can inhibit the growth of STTG1 cells by inducing oxidative stress-mediated apoptosis (Rajan et al., 2016). In addition, MIC-1 can suppress the growth of Caco-2 colon cancer cells and HepG2 liver cancer cells, with IC_50_ values of 45 μg/ml and 60 μg/ml, respectively (Maiyo et al., 2016). Similarly, MIC-1 inhibits the growth of SH-SY5Y human neuroblastoma cells in a time- and concentration-dependent manner, and induces SH-SY5Y cell apoptosis and cell cycle arrest by increasing the mRNA and protein levels of P53, P21, Bax, caspase-3, and caspase-9, and blocking the nuclear translocation of NF-κB (Cirmi et al., 2019). In the present study, we demonstrated that MIC-1 could inhibit the proliferation and migration of 786-O and 769-P RCC cells, while also inducing their apoptosis and cell cycle arrest. Additionally, we also clarified the underlying mechanism, which involved MIC-1 inhibition of PTP1B-mediated activation of the Src/Ras/Raf/ERK signaling pathway.

Traditionally, research on PTP1B has mainly focused on its regulatory role in glucose and lipid metabolism. However, it is now clear that PTP1B is closely related to the occurrence and development of several tumors (Yip et al., 2010). Studies have shown that the *PTP1B* gene is abnormally highly expressed in ovarian cancer, gastric cancer, prostate cancer, and breast cancer and is associated with poor prognosis (Hoekstra et al., 2016; Wang et al., 2018; Xu et al., 2019; Yu et al., 2019). High PTP1B expression can increase Ras activity, thereby increasing the risk of tumorigenesis (Dubé et al., 2004). In addition, PTP1B can also promote tumor cell growth, migration, and invasion by regulating Src activity. For example, PTP1B expression is upregulated in colon cancer, and this high expression can inhibit the phosphorylation of Src Tyr529, which leads to the rapid proliferation of cancer cells *in vitro* and the acceleration of tumor growth in mice (Zhu et al., 2007). In breast cancer cells, high PTP1B expression is associated with Src activation and increased pseudopodia formation; however, inhibiting PTP1B can delay the formation of breast tumors and reduce the risk of breast cancer lung metastasis through the suppression of Src/Ras/ERK and AKT signaling pathways (Julien et al., 2007; Cortesio et al., 2008; Arias-Romero et al., 2009). Similarly, in nonsmall cell lung cancer and gastric cancer, the expression of PTP1B is upregulated, leading to increased proliferation and metastasis of cancer cells through the activation of the Src/Ras/ERK and PI3K/AKT signaling pathways; however, when PTP1B is knocked down, cancer cell growth is inhibited, and cell cycle arrest and apoptosis are induced (Liu et al., 2015; Wang et al., 2015). These results indicate that PTP1B has potential as a key anticancer target. In this study, we revealed that MIC-1 inhibits the Src/Ras/Raf/ERK signaling pathway by suppressing the activity of PTP1B, thereby preventing the growth and migration of RCC cells; nevertheless, further investigation is needed to fully elucidate the relationship between PTP1B and the occurrence and development of RCC.

It is known that PTP1B is related to the occurrence and development of various cancer cells such as ovarian cancer, stomach cancer, prostate cancer, breast cancer, colon cancer, and non-small cell lung cancer. In this study, we found that MIC-1 could significantly inhibit the activity of PTP1B and inhibit the growth of a variety of cancer cells. Further research found that MIC-1 could inhibit the growth and migration of RCC cells by regulating the PTP1B-dependent Src/Ras/Raf/ERK signaling pathway. However, whether the growth inhibitory effect of MIC-1 on other cancer cells is related to the regulation of the activity of the PTP1B signaling pathway, and whether the effect of MIC-1 on RCC cells is specific is still unknown. Therefore, we studied the regulatory effect of MIC-1 on PTP1B-related signaling pathways in different cancer cells: HCT-116 cells and Hep-G2 cells are sensitive to the growth inhibition of MIC-1, and A431 cells that is insensitive to the effects of MIC-1. We found that, MIC-1 had no effect on the PTP1B-dependent Src/Ras/Raf/ERK signaling pathway in HCT-116 cells, Hep-G2 cells, and A431 cells. These results indicate that MIC-1 specifically acts on the PTP1B-related Src/Ras/Raf/ERK signaling pathway to inhibit the growth and migration in RCC cells. As for the mechanism of MIC-1 inhibiting the growth of HCT-116 cells and Hep-G2, as well as the relationship between MIC-1 and PTP1B and different cancer cells still need to be further explored.

## Data Availability

The original contributions presented in the study are included in the article/Supplementary Material, further inquiries can be directed to the corresponding authors.
