# Supplementary material for: Isothiocyanate From Moringa oleifera Seeds Inhibits the Growth and Migration of Renal Cancer Cells by Regulating the PTP1B-dependent Src/Ras/Raf/ERK Signaling Pathway
Source: Front Cell Dev Biol. 2022 Jan 4;9:790618. doi: 10.3389/fcell.2021.790618 (PMC8764249; doi:10.3389/fcell.2021.790618)
Supplement: Supplementary file 1 [file Table1.DOCX]

**Table 1 MIC-1 binding target screened by PharmMapper server**

| Number | Name | Z’-score |
| --- | --- | --- |
| 1 | Platelet glycoprotein Ib alpha chain | 4.48622 |
| 2 | Histo-blood group ABO system transferase | 4.20056 |
| 3 | CD209 antigen | 3.65447 |
| 4 | Peptidyl-prolyl cis-trans isomerase FKBP1B | 3.48611 |
| 5 | Tyrosine-protein phosphatase non-receptor type 1 | 3.4148 |
| 6 | Glucosamine-6-phosphate isomerase | 3.15963 |
| 7 | Phenylalanine-4-hydroxylase | 3.04667 |
| 8 | Proto-oncogene serine/threonine-protein kinase Pim-1 | 3.0145 |
| 9 | Beta-hexosaminidase subunit beta | 2.85659 |
| 10 | Sorbitol dehydrogenase | 2.75548 |
